# Preliminary Evaluation of the Anticancer Activity of a PGK1 Small Molecule Inhibitor in Ishikawa Endometrial Cancer

**DOI:** 10.3390/ijms27219641

**Published:** 2026-10-28

**Authors:** Haijing Cao, Shengyu Xue, Man Xue, Fang Li, Linglin Feng, Wei Zheng, Hong Ding, Ting Zhang

**Affiliations:** 1 NHC Key Lab of Reproduction Regulation, Shanghai Engineering Research Center of Reproductive Health Drug and Devices, Shanghai Institute for Biomedical and Pharmaceutical Technologies, Shanghai 200237, China; caohaijing1341@163.com (H.C.); manxue91627@163.com (M.X.); lfdalle@163.com (F.L.); fenglinglinxin@163.com (L.F.); zhengwei726@126.com (W.Z.); 2 School of Chinese Materia Medica, Nanjing University of Chinese Medicine, Nanjing 210023, China; xueshengyu@simm.ac.cn; 3 State Key Laboratory of Drug Research, Shanghai Institute of Materia Medica, Chinese Academy of Sciences, 555 Zuchongzhi Road, Shanghai 201203, China

**Keywords:** endometrial cancer, glycolysis, PGK1 inhibitor, NG52, activity, migration, apoptosis

## Abstract

To investigate the roles of a phosphoglycerate kinase 1 (PGK1) small-molecule inhibitor in Ishikawa endometrial cancer. The role of a PGK1 small molecule inhibitor in endometrial cancer was identified by cell viability, transwell, cell cycle, and apoptosis analyses. The expression levels of PGK1 in cancer cells treated with various concentrations of the PGK1 small molecule inhibitor at 48 h and 72 h were determined via Western blotting. Xenograft mouse models were established through subcutaneous tumor transplantation, and immunofluorescence analysis of PGK1 and apoptosis in tumor tissue was performed. The results of the cell counting kit-8 (CCK-8) assay revealed that NG52 inhibited the activity of human endometrial carcinoma Ishikawa, HEC-1-A, and HEC-1-B cells in a concentration-dependent manner. Moreover, NG52 significantly reduced the migration ability of the Ishikawa cell line in a dose-dependent manner. Flow cytometry (FCM) demonstrated that NG52 affected the survival of endometrial carcinoma cells by arresting the cell cycle at the G1 phase and inducing apoptosis in a dose-dependent manner. Western blot analysis also revealed that the apoptosis-inducing effects of NG52 were accompanied by a reduction in PGK1 expression after 48 h and 72 h of NG52 treatment. Moreover, NG52 partially suppressed tumor growth in a human Ishikawa xenograft model, and transferase dUTP nick end labeling (TUNEL) showed an increasing trend of NG52-treated tumor tissue apoptosis, with a decreasing trend in PGK1 expression in the treatment group. NG52, a PGK1 small molecule inhibitor, can serve as a structural basis for developing potential anti-endometrial carcinoma agents. The findings are limited to the preliminary preclinical activity of NG52, and further mechanistic, pharmacologic, efficacy, and safety studies are warranted before improved PGK1 inhibitors can proceed to clinical translation.

## 1. Introduction

Endometrial cancer is one of the three major gynecological malignant tumors. In recent years, the incidence of endometrial cancer has increased annually [1,2,3]. However, the detailed molecular mechanisms involved in endometrial carcinoma malignancy and relapse remain elusive [4,5]. Body mass index and the waist-to-hip ratio are reportedly associated with increased cancer risk in premenopausal women. Disruption of glucose metabolism may be one of the causes of this disease [6].

Recent studies have shown that the glycolysis pathway plays an important role in the occurrence and development of malignant tumors [7]. The transformation of normal cells into malignant tumor cells is accompanied by the remodeling of metabolic pathways. Abnormal glucose metabolism is an important characteristic of tumor cells. The proliferation rate of tumor cells in malignant tumors exceeds their energy supply. The excessive consumption of oxygen and nutrients leads to a tumor microenvironment of low oxygen, low glucose and acidity, which is more obvious in solid tumors. The main approach to enhance glycolysis in tumor cells is to adjust the specific enzymes in the glycolysis pathway to increase their activity and overexpression [8,9,10].

Phosphoglycerate kinase 1 (PGK1) is the first enzyme in glycolysis that generates a molecule of ATP in the conversion of 1,3-bisphosphoglycerate (1,3-BPG) to 3-phosphoglycerate (3-PG) [11,12]. PGK1 is overexpressed in most cancers, such as neuroblastoma [13], glioma [14,15], oral squamous cell carcinoma [16], non-small cell lung cancer [17], breast cancer [18], gastric cancer [19], pancreatic cancer [20], liver cancer [21], colorectal cancer [22], clear cell renal cell carcinoma [23], and ovarian cancer [24]. High expression of PGK1 promotes the growth, metastasis, invasion and drug resistance of cancer cells. Some studies have shown that PGK1 overexpression is also significantly related to the occurrence of endometrial cancer [25,26].

NG52 has been reported to be a specific small-molecule inhibitor of PGK1 and has been shown to have anticancer activity in glioblastoma, ovarian cancer and clear cell renal cell carcinoma [27,28,29]. Here, it was used as a small-molecule probe in experiments to determine the role of PGK1 inhibitors in endometrial cancer.

## 2. Results

### 2.1. Cell Viability Assay

**Figure 1:**
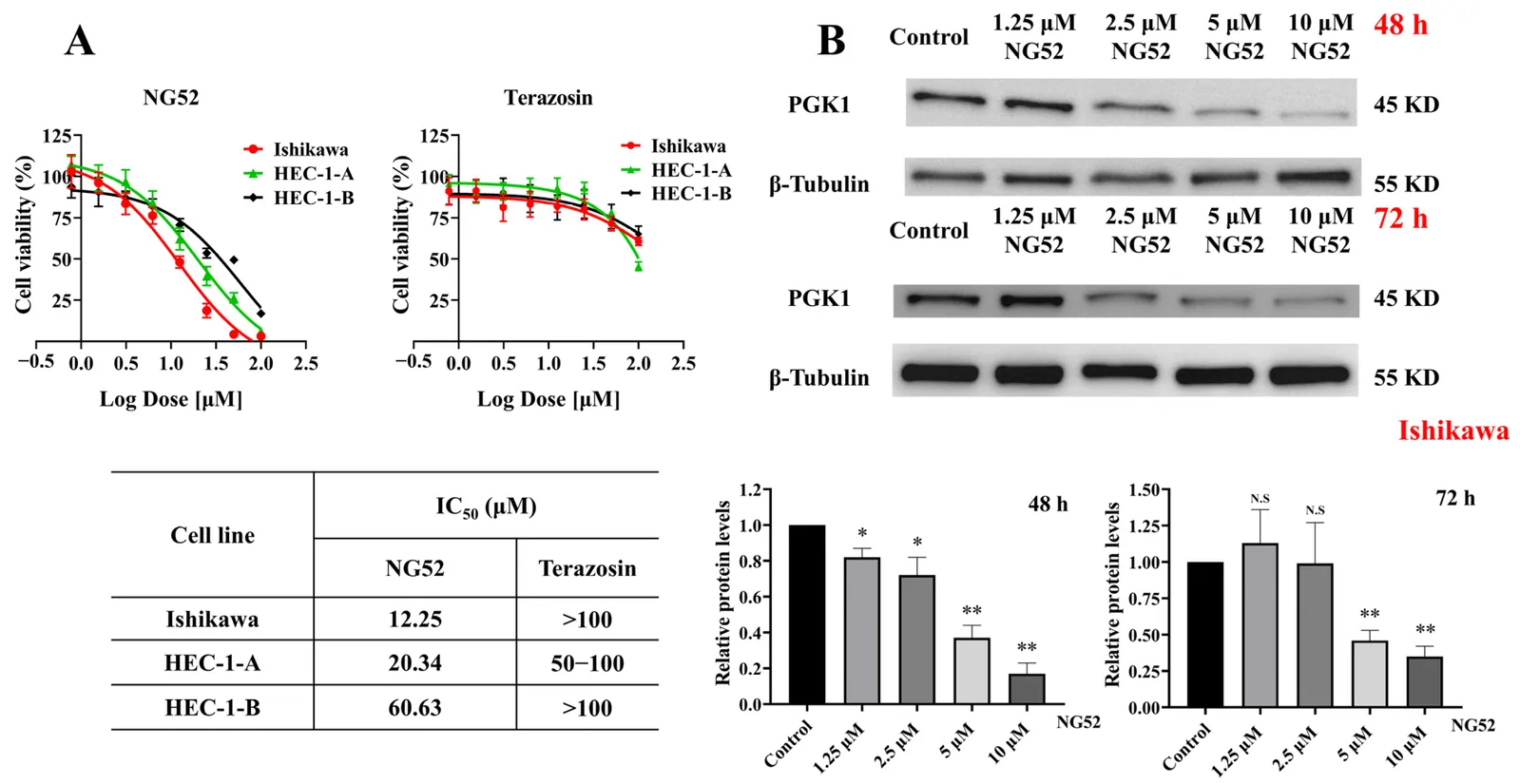
Effect of NG52 on endometrial cancer cells. (**A**) Three human endometrial cell lines (Ishikawa, HEC-1-A, and HEC-1-B) were treated with various concentrations of NG52 or terazosin for 72 h to measure cell viability and IC_50_ values. (**B**) PGK1 protein expression in an endometrial cancer cell line (Ishikawa) after 48 h and 72 h of NG52 treatment. Data are shown as mean ± SD based on three independent experiments. N.S., not significant; * *p* < 0.05; ** *p* < 0.01, compared with the negative control group.

Three human endometrial cell lines, including Ishikawa, HEC-1-A and HEC-1-B cells, were treated with various concentrations of NG52 and terazosin for 72 h. The goal was to test cell viability and determine the half-maximal inhibitory concentration (IC_50_). The average IC_50_ values of NG52 were 12.25, 20.34, and 60.63 μM for Ishikawa, HEC-1-A and HEC-1-B cells, respectively. Moreover, for terazosin, based on the concentration–response data, the IC_50_ exceeded 100 μM in Ishikawa and HEC-1-B cells. In HEC-1-A cells, viability was 78.34 ± 2.12% at 50 μM, but fell below the 50% threshold at 100 μM (45.64 ± 2.66%), indicating that the IC_50_ lies between 50 and 100 μM; because the lower plateau of the inhibition curve was not reached within the tested range, this value could not be precisely determined (Figure 1A). Terazosin did not seem to have an antiproliferative effect on endometrial cancer cell lines.

### 2.2. Western Blot

The results revealed that the expression levels of PGK1 significantly decreased after 48 h and 72 h of NG52 treatment in a dose-dependent manner (Figure 1B). After 48 h of treatment, PGK1 protein levels were significantly reduced at 1.25 μM (*p* = 0.0238) and 2.5 μM (*p* = 0.0377), with more pronounced decreases observed at 5 μM (*p* = 0.0038) and 10 μM (*p* = 0.0016), where PGK1 expression fell to approximately 0.17-fold of the control level. A similar pattern was observed after 72 h of treatment: PGK1 expression was not significantly altered at 1.25 μM (*p* = 0.4366) or 2.5 μM (*p* = 0.9572), but was significantly decreased at 5 μM (*p* = 0.0055) and 10 μM (*p* = 0.0038), indicating that NG52 downregulates PGK1 in a concentration-dependent manner in Ishikawa cells (That experimental details are provided in the Supplementary Materials).

### 2.3. Migration of Endometrial Cancer Cells

**Figure 2:**
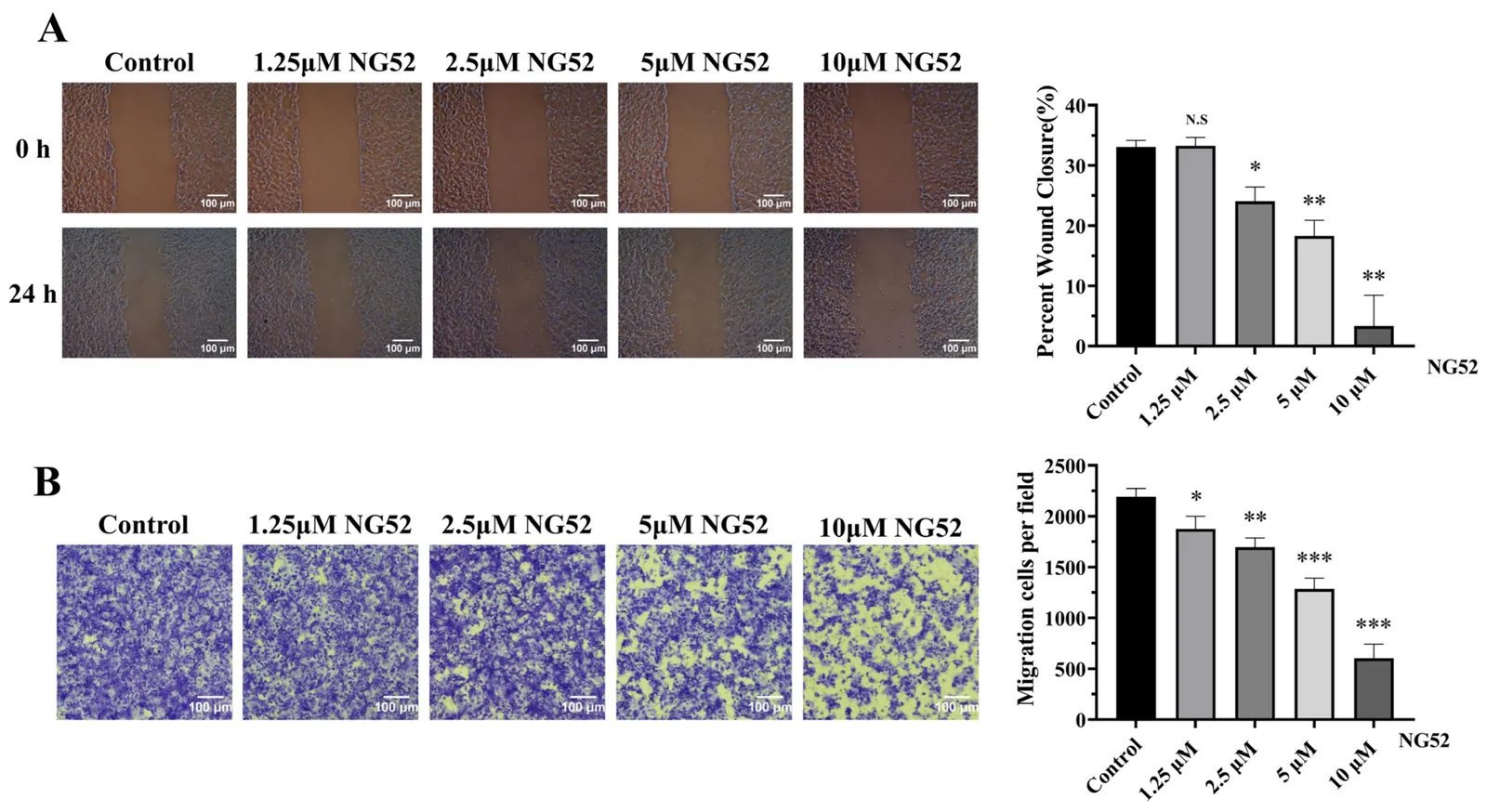
Migration of endometrial cancer cells following 24 h of NG52 treatment. (**A**) NG52 significantly reduced the migration ability of Ishikawa cells in a dose-dependent manner. * *p* < 0.05, ** *p* < 0.01, compared with the negative control group. (**B**) NG52 significantly inhibited the migration of drug-treated cells. Data are shown as mean ± SD based on three independent experiments. N.S., not significant; * *p* < 0.05; ** *p* < 0.01; *** *p* < 0.001, compared with the negative control group.

NG52 significantly reduced the migration ability of Ishikawa cells in a dose-dependent manner. NG52 significantly inhibited cell fusion at the scratch site under a microscope at the 0 and 24 h time points (Figure 2A). While the scratch wounds of untreated control cells were partially closed after 24 h, wound closure was progressively impaired with increasing NG52 concentrations. Quantification revealed that percent wound closure was comparable between the control and 1.25 μM NG52 groups (*p* = 0.8569), whereas 2.5 μM (*p* = 0.0114), 5 μM (*p* = 0.0042), and 10 μM (*p* = 0.0075) NG52 significantly reduced wound closure compared with the control. Notably, treatment with 10 μM NG52 nearly abolished wound closure (from approximately 33.25% in control cells to approximately 3.34%).

Moreover, NG52 significantly inhibited the migration of drug-treated cells after 24 h of treatment (Figure 2B). Moreover, NG52 significantly inhibited the migration of drug-treated cells after 24 h of treatment. Compared with the negative control (approximately 2190 migrated cells per field), NG52 treatment reduced the number of migrated cells in a concentration-dependent manner, with statistically significant decreases observed at 1.25 μM (*p* = 0.0276), 2.5 μM (*p* = 0.0023), 5 μM (*p* = 0.0004), and 10 μM (*p* = 0.0003). The highest concentration, 10 μM, reduced cell migration by approximately 72.39% relative to the control. Taken together with the wound-healing results (Figure 2A), these data indicate that NG52 suppresses the migratory capacity of endometrial cancer cells in a dose-dependent manner.

### 2.4. Cell Cycle Progression and Cell Apoptosis

**Figure 3:**
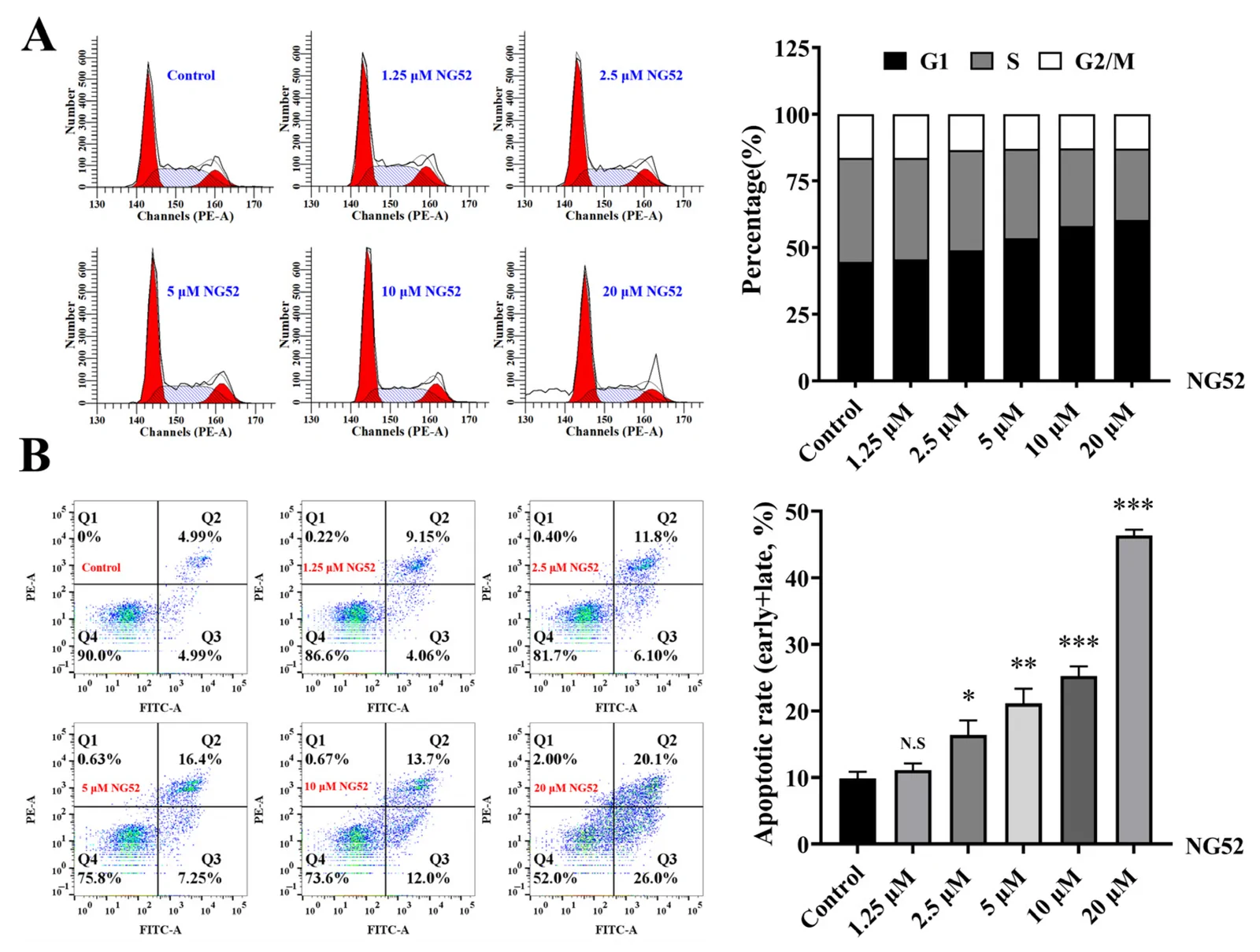
Cell cycle progression and cell apoptosis of Ishikawa cell lines after NG52 treatment. (**A**) The cell cycle of tumor cells was blocked by NG52 at the G1 phase. (**B**) NG52 promoted tumor cell apoptosis. Representative flow cytometry plots from one of three independent experiments are shown. The quantitative results are presented as mean ± SD of three independent experiments (*n* = 3). N.S., not significant; * *p* < 0.05; ** *p* < 0.01; *** *p* < 0.001, compared with the negative control group.

The experimental results were analyzed via ModFit software 3.1, and high doses of NG52 were found to block the cell cycle of tumor cells in the G1 phase, thereby inhibiting cell activity (Figure 3A). Compared with the negative control, NG52 promoted tumor cell apoptosis in a dose-dependent manner (Figure 3B). The increase did not reach statistical significance at 1.25 μM (*p* = 0.2024), whereas significantly higher apoptotic rates were observed at 2.5 μM (*p* = 0.0224), 5 μM (*p* = 0.0056), 10 μM (*p* = 0.0002), and 20 μM (*p* = 0.0001). Collectively, these results indicate that NG52 induces apoptosis of endometrial cancer cells in a concentration-dependent manner.

### 2.5. Nude Mouse Xenograft Tumor Model

**Figure 4:**
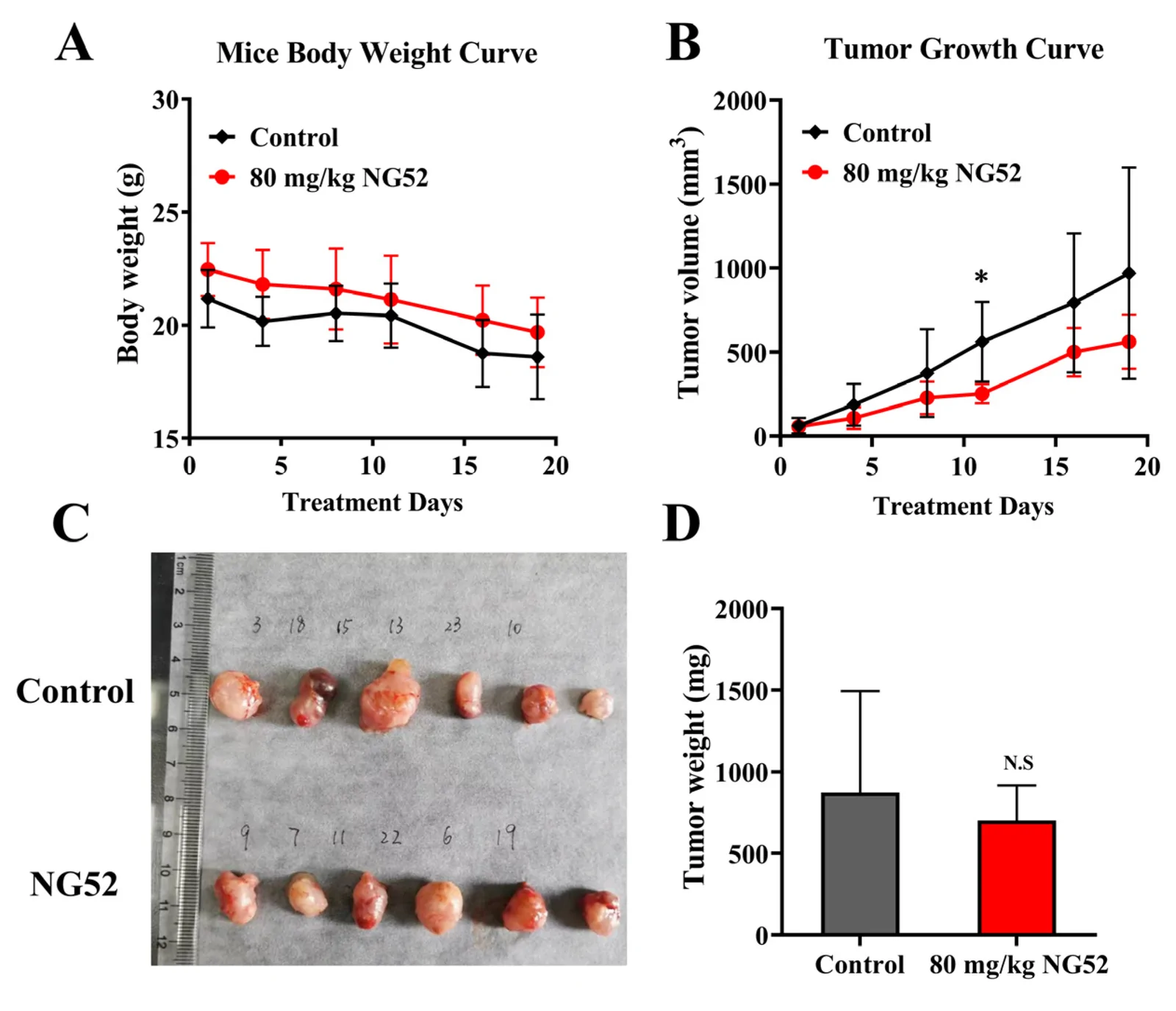
Body weight curves of nude mice and growth curves of Ishikawa cell xenograft tumors after drug administration (**A**,**B**) and tumor morphology and weight (**C**,**D**). Data are shown as mean ± SD. N.S., not significant; * *p* < 0.05, compared with the negative control group.

Twenty-three female nude mice were subcutaneously inoculated with Ishikawa cells. At week 3 after inoculation, no tumor mass was observed in 8 mice. A total of 12 mice had tumors that met the volume criteria for the study. The tumorigenicity rate of Ishikawa cells after 21 days of inoculation was 65.2%, and the rate after 28 days was 78.2%. In this study, the tumor volume reached the experimental standard at 21 days after inoculation, and the tumors were divided into two groups, each with 6 nude mice. Following 3-week treatment, a slight gradual decline in body weight was observed in both test and negative control groups, and there was no statistically significant difference between them (Figure 4A). Compared with the negative control group, there was a significant difference in tumor volume on day 11 (*p* = 0.0226, Figure 4B). The best T/C ratio was observed on day 11, with a T/C ratio of 45.33%, and the TGI was 54.67%. There was still an apparent inhibition trend at the end of the experiment (day 19), with a TGI of 38.75%. At the experimental endpoint, tumor tissues of each group were excised, photographed and weighed; however, there was no statistically significant difference in tumor weight (Figure 4C,D).

### 2.6. TUNEL Analysis and Immunofluorescence

**Figure 5:**
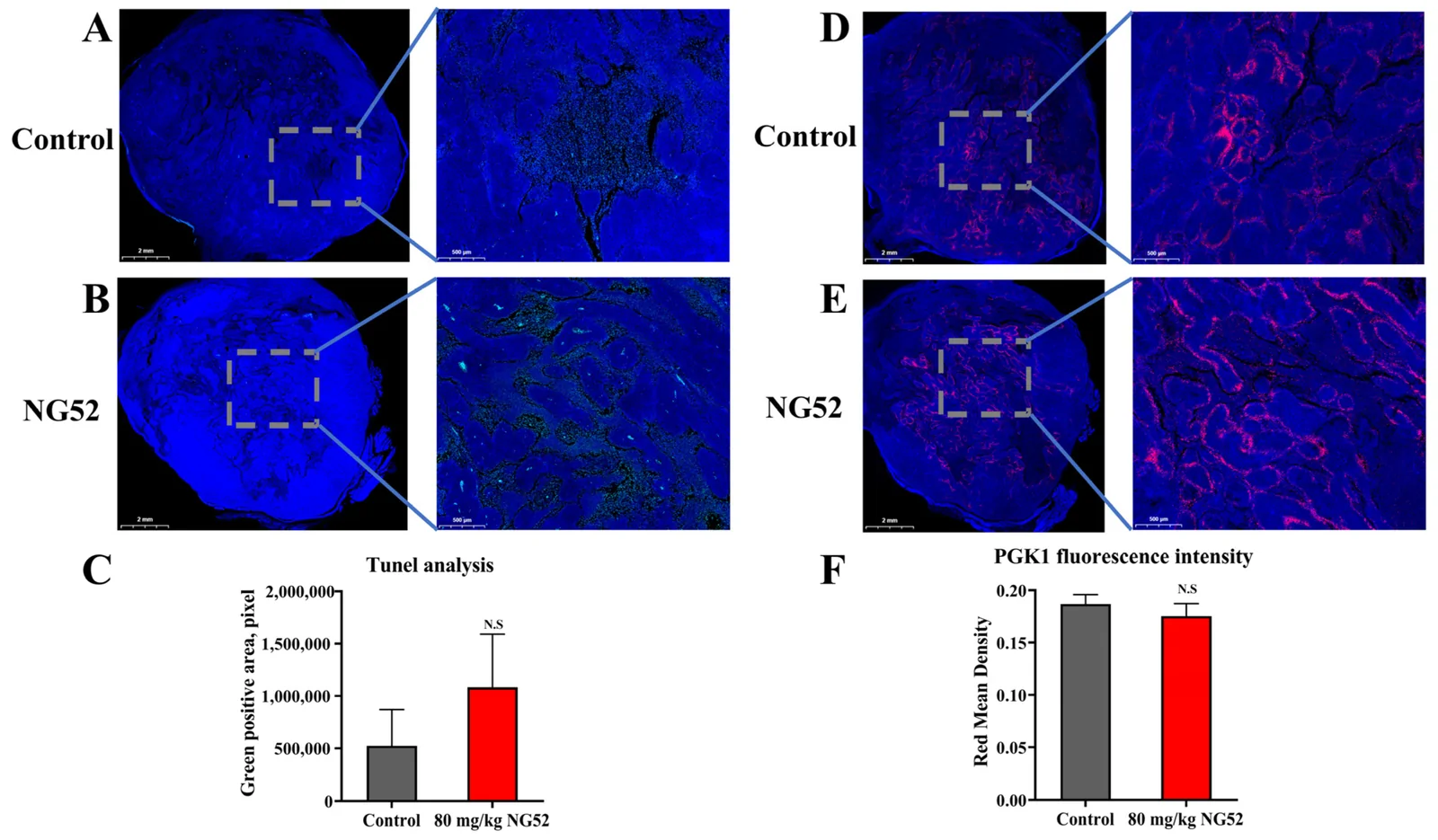
TUNEL analysis and immunofluorescence of PGK1. (**A**–**C**) Representative images of tumor tissues subjected to TUNEL staining. The green-positive area of each tumor stained with TUNEL was statistically analyzed. (**D**–**F**) The expression level of PGK1 is presented as the red mean density in both groups. Data are shown as mean ± SD. N.S., not significant; there was no statistically significant difference between the two groups.

TUNEL analysis was performed by comparing the green-positive area to that of the negative control group. The results revealed that, in the treatment group, the green-positive area showed an increasing trend, which indicated the occurrence of NG52-treated tumor tissues undergoing apoptosis, but the difference was not statistically significant (*p* = 0.3402, Figure 5A–C). PGK1 was expressed in both the control group and the treatment group, with a decreasing trend in PGK1 expression in the treatment group compared with the control group (*p* = 0.3921, Figure 5D–F).

## 3. Discussion

The main treatment for endometrial cancer is surgical resection, supplemented by radiotherapy and drug therapy. At present, commonly used hormone drugs include progesterone, gonadotropin-releasing hormone analogs, aromatase inhibitors, and chemotherapy drugs such as paclitaxel, carboplatin, cisplatin, and doxorubicin. Chemotherapy resistance is the main problem faced in clinical practice. So far, few novel therapeutic targets and innovative drugs are available for endometrial carcinoma.

PGK1 plays an important role in cancer and is also associated with chemoradiotherapy resistance and poor prognosis in cancer patients. PGK1 promotes tumor growth, invasion, metastasis, and drug resistance through multiple signaling pathways. Under conditions of low oxygen and amino acid deficiency, the overexpression or posttranslational modification of PGK1 can activate the metabolic enzyme activity of PGK1, promote sugar metabolism in tumor cells, provide more energy and biomass for tumor growth, and increase cell tolerance to reactive oxygen species. Moreover, the protein kinase PGK1 can assist in DNA damage repair and synthesis and induce cellular autophagy, thereby promoting tumor drug resistance [30,31]. PGK1 is suggested as a potential biomarker for the early diagnosis and prognosis of breast cancer and hepatocellular carcinoma [32,33]. Some clinical research data have also revealed a significant correlation between drug resistance and the overexpression of PGK1 in endometrial cancer [25,26].

On the basis of the roles of PGK1 in tumors, small molecule inhibitors of PGK1 have been developed to identify suitable PGK1 inhibitors for the treatment of tumors. Some studies have indicated that low-concentration terazosin activates PGK1, elevating ATP generation to stimulate HSP90 and inhibit apoptosis. By contrast, terazosin serves as a PGK1 inhibitor when the concentration exceeds 2.5 µM in vitro. The structure of terazosin has been modified to identify small-molecule compounds with stronger inhibitory effects on PGK1 [34,35,36,37]. This study examined the inhibitory effect of terazosin on endometrial cancer cells. The results showed that no apparent antiproliferative activity was observed even at concentrations exceeding 100 µM, which might indicate that small molecules derived from TZ structural modification may have limited applicability in endometrial carcinoma therapy.

As a validated inhibitor of PGK1, NG52 has been shown to have antiproliferative effects on various types of tumors, including glioblastoma, ovarian cancer, and clear cell renal cancer [27,28,29]. In our studies, NG52 showed significant inhibition against the endometrial carcinoma Ishikawa cell line at around 10 μM, whereas no marked inhibitory effect was observed for the other two cell lines. However, as NG52 acts on the metabolic pathways of tumor cells, results from the CCK-8 assay may produce false-positive signals, suggesting that the inhibitory effect of NG52 on tumor cell proliferation might not be as strong as indicated by CCK-8 data. Importantly, even when cells merely undergo metabolic suppression instead of full cell death, their cellular activity is largely impaired. Treatment combining NG52 with chemotherapeutic drugs sensitized these metabolically damaged cells to chemotherapy. We have conducted combination assays of NG52 and several chemotherapeutic agents, revealing an approximately 10–30% reduction in tumor cell viability after combined treatment. Western blot analysis revealed a significant decrease in PGK1 expression in a dose-dependent manner after 48 h and 72 h of treatment, indicating that the viability-inhibitory effect of NG52 on endometrial cancer cell lines is accompanied by the changes in PGK1 expression. Together with the reported direct, ATP-competitive inhibition of PGK1 by NG52 (IC_50_ 2.5 μM) and the reduced proliferation, clonogenicity, and NG52 sensitivity of PGK1-knockdown cells in glioma [27], these findings support a PGK1-mediated component of NG52 activity in endometrial cancer cells. In the present study, however, PGK1 involvement was inferred from protein abundance alone, without activity or target-engagement measurements, correlation analysis, rescue experiments, or glycolytic readouts; the findings therefore remain associative and hypothesis-generating, with mechanistic support derived from prior reports. In addition to in vitro experiments, NG52 can inhibit the migration of tumor cells and promote cell apoptosis, and in vivo experiments have also shown that NG52 can inhibit the growth of tumor tissue at 80 mg/kg without affecting animal weight gain. However, immunofluorescence detection of PGK1 in animal experiments revealed no significant difference between the two groups, suggesting that other factors in the in vivo microenvironment may modulate its antitumor growth effect. Apoptosis was documented by complementary assays based on different molecular events (Annexin V-FITC/PI staining and TUNEL staining). Both methods showed that NG52 increased apoptosis. Protein-level apoptosis markers such as cleaved caspase-3, cleaved PARP or the BAX/BCL-2 ratio were not assessed, and the specific apoptotic pathway involved therefore remains to be defined. In addition, NG52 lacks strong PGK1 selectivity and has a relatively high therapeutic dose; therefore, it is unlikely to be developed as a therapeutic drug for endometrial cancer. Nevertheless, structural modification of the compound may afford small molecules with better activity in the treatment of endometrial carcinoma.

In summary, NG52 has a potential inhibitory effect on endometrial cancer and can be used as a structural basis for further research. In the future, better-activity PGK1 small-molecule inhibitors may be found through structural modification of these compounds.

## 4. Materials and Methods

### 4.1. Compounds and Reagents

NG52 (CAS: 212779-48-1, purity ≥ 98%) was obtained from Beijing Paitong Biotechnology Co., Ltd. (Beijing, China). Terazosin hydrochloride dihydrate (CAS:70024-40-7, purity ≥ 98%) was obtained from Aladdin Biochemical Technology Co., Ltd. (Shanghai, China). A Cell Counting Kit-8 (CCK-8) was purchased from Dojindo Laboratories (Tokyo, Japan). An Annexin V-FITC/PI Apoptosis Detection Kit and a Cell Cycle and Apoptosis Analysis Kit were purchased from Yeasen Biotechnology Co., Ltd. (Shanghai, China). Trypsin-EDTA (0.25%) was purchased from Gibco Chemical Co., Ltd. (Carlsbad, CA, USA). Phosphate-buffered saline (PBS) and Dulbecco’s modified Eagle’s medium (DMEM) were purchased from HyClone (Healthcare Life Sciences, South San Francisco, CA, USA).

### 4.2. Cell Culture

Human endometrial carcinoma Ishikawa cells (MZ-0207) and specific medium (MCM-0283) were purchased from Ningbo Mingzhou Biotechnology Co., Ltd. (Ningbo, China). HEC-1-A and HEC-1-B cells were kindly donated by Professor Luo’s laboratory at the Shanghai Institute of Materia Medica, Chinese Academy of Sciences. These two cell lines were stored in our laboratory and cultured in EMEM supplemented with 10% fetal bovine serum (Gibco) and 1% penicillin/streptomycin (HyClone). The cells were propagated in a humidified atmosphere with 5% CO_2_ at 37 °C.

### 4.3. Cell Viability Assay

A CCK-8 assay was carried out to measure the cytotoxicity of NG52 to the endometrial cancer cell lines Ishikawa, HEC-1-A and HEC-1-B. The logarithmic phases of endometrial cancer Ishikawa, HEC-1-A and HEC-1-B cells were plated onto a 96-well culture plate at a volume of 100 µL per well and incubated at 37 °C overnight. NG52 was dissolved in dimethyl sulfoxide (DMSO) to a 20 mM stock solution and diluted in complete culture medium to the final working concentrations. The cells were subsequently incubated with NG52 at 100, 50, 25, 12.5, 6.25, 3.125, 1.56 and 0.78 μM for 72 h. Each concentration had three replicates. The final DMSO concentration did not exceed 0.5% (*v*/*v*). After 72 h, different concentrations of the test substance were removed from each well, followed by the addition of 100 µL of fresh culture medium and 10 µL of CCK-8 and incubation for 2 h before absorbance measurement. The absorbance at 450 nm was detected by scanning with a Multiskan™ FC Microplate Reader (Thermo Fisher Scientific Inc., Shanghai, China). The IC_50_ values were obtained solely by fitting the dose–response curves (four-parameter variable-slope nonlinear regression, GraphPad Prism 8).

### 4.4. Western Blot

The cells were collected and lysed with precooled RIPA lysis buffer, and the protein concentration was quantified via the BCA method. The proteins were separated on a 12.5% SDS-PAGE gel and transferred to a polyvinylidene difluoride (PVDF) membrane (Millipore, Billerica, MA, USA). The membrane was blocked with 5% milk for 2 h. Next, the membrane was incubated with the following primary antibody at 4 °C overnight: anti-PGK1 (1:2000; ab199438; Abcam, Shanghai, China). β-Tubulin (1:10,000; 30303ES50; Yeasen, Shanghai, China) was used as an internal control. The membrane was incubated with a secondary antibody (1:50,000, ab205718; Abcam, Shanghai, China) for 2 h at room temperature and visualized with an enhanced chemiluminescence (ECL) reagent (36208ES60; Yeasen, Shanghai, China).

### 4.5. Wound-Healing Assay

A suspension of Ishikawa cells grown at the logarithmic phase was transferred to 6-well plates and incubated. After Ishikawa cells grew to confluence in a 6-well plate, they were cultured overnight in serum-free medium. A 200 µL pipette tip was then used to create a wound by passing over the well, and the detached cells were removed by washing with PBS. Three replicates were set up for each group, including a control group and different concentrations of NG52. At 0 h and 24 h, the cell confluence at the wound site was observed under a microscope, and photos were taken and recorded, including the distance between the adherent cells on both sides of the wound.

### 4.6. Transwell Assay

Three hundred microliters of a single-cell suspension (1 × 10^6^ cells) diluted with serum-free medium was added to the upper chamber, and 500 µL of medium containing 20% fetal bovine serum was added to the lower chamber. After 24 h of incubation, the cells were fixed with 4% paraformaldehyde for 20 min and stained with crystal violet for 60 min. Pictures were taken with a microscope.

### 4.7. Cell Cycle Analysis

After 72 h of treatment, the cells were collected, washed twice with PBS and fixed in 70% ethanol at 4 °C overnight. Then, the cells were collected and washed once and incubated in 400 μL of PBS with 50 μg/mL PI, 50 μg/mL RNase A and 0.2% Triton 100 for 30 min at 37 °C. The cell cycle distribution data were collected with an LSR Fortessa cytometer (Becton Dickinson, New York, NY, USA). The data were analyzed via ModFit software v3.1 (Verity Software House, Topsham, ME, USA).

### 4.8. Apoptosis Analysis

After 72 h of treatment, the cells were collected and washed twice with PBS. Then, the cells were suspended in 500 μL of binding buffer and stained with both 5 μL of PI staining solution and 10 μL of annexin V-FITC, which were gently mixed and incubated at room temperature in the dark for 10 min. The samples were detected by flow cytometry within 1 h after the reaction was completed. The data were analyzed via FlowJo software v10.6.2 (FLOWJO, LCC, Ashland, OR, USA).

### 4.9. Xenograft Tumor Models

Twenty-three female BALB/c nude mice aged 4–5 weeks were obtained from Shanghai Slike Experimental Animal Co. (Shanghai, China). The present animal experiments were conducted according to protocols approved by the animal ethics committee of the Shanghai Institute for Biomedical and Pharmaceutical Technologies (Permit Number: 2020-08). Ishikawa cells (1 × 10^6^/100 μL) were subcutaneously injected into the left flank of each mouse. After the tumors reached a volume of 50–100 mm^3^, the mice were randomly assigned to the negative control group (*n* = 6) or the treatment group (*n* = 6) according to tumor volume. The mice in the treatment group were administered NG52 at 80 mg/kg, 5 times weekly for 3 consecutive weeks by gavage, and those in the control group were treated with vehicle only (0.5%CMC). The tumor diameter and mouse body weights were measured twice a week for three weeks. The tumor volume was calculated via the following formula: [1/2× (width)^2^ × length]. After the mice were sacrificed via excessive CO_2_ inhalation anesthesia, the tumors were removed and fixed with 4% paraformaldehyde for further analysis. All procedures were performed in strict accordance with the recommendations of the Guide for the Care and Use of Laboratory Animals of the National Institutes of Health. The relative tumor volume (RTV) was calculated as [volume at each measurement/volume at initial treatment]. T/C was calculated as [the mean RTV of the treatment group/the mean RTV of the control group] ×100%, which was used to assess the antitumor efficacy of NG52. Tumor Growth Inhibition (TGI) was calculated as 100%—(T/C).

### 4.10. TUNEL Analysis

The tumors were excised, fixed with formalin and then embedded in paraffin. After the sections were cut according to the manufacturer’s protocol, their apoptosis was assessed via a transferase dUTP nick end labeling (TUNEL) system (Wuhan Service Biotechnology Co., Ltd., Wuhan, China). The images were obtained with a Nikon Eclipse C1 Ortho-fluorescence microscope. The data were analyzed via Aipathwell v2^®^ software (Wuhan Service Biotechnology Co., Ltd., Wuhan, China).

### 4.11. Immunofluorescence

Immunofluorescence analysis was performed on serial formalin-fixed, paraffin-embedded tissue sections. The sections were first deparaffinized in xylene and then rehydrated. The antigens were retrieved by heat-induced antigen retrieval for 20 min in citric acid buffer, followed by blocking with 2% BSA for 30 min and then incubation with a primary antibody (anti-PGK1, 1:250, AB199438, Abcam, Cambridge, UK) at 4 °C overnight. The sections were subsequently incubated for 2 h at room temperature with CY3-labeled goat anti-rabbit IgG secondary antibodies (Wuhan Service Biotechnology Co., Ltd., Wuhan, China). The images were obtained with a Nikon Eclipse C1 Ortho-fluorescence microscope. The data were analyzed via Aipathwell^®^ software (Wuhan Service Biotechnology Co., Ltd., Wuhan, China).

### 4.12. Statistical Analysis

Data analyses included calculations of group means and standard deviation (SD). All data are presented as mean ± SD unless otherwise stated. The count data were analyzed via the chi-square test and Fisher’s exact probability test, and the measurement data were analyzed via Student’s *t*-test. *p* < 0.05 was considered statistically significant.

## Data Availability

Data are available upon request.

## Supplementary Materials

The following supporting information can be downloaded at: https://www.mdpi.com/article/10.3390/ijms27219641/s1.
