# Supplementary material for: Preliminary Evaluation of the Anticancer Activity of a PGK1 Small Molecule Inhibitor in Ishikawa Endometrial Cancer
Source: Int J Mol Sci. 2026 Oct 28;27(21):9641. doi: 10.3390/ijms27219641 (PMC13763334; doi:10.3390/ijms27219641)
Supplement: Supplementary file 1 [file ijms-27-09641-s001.zip › ijms-4576239-supplementary.pdf]

WB-48h-Test1

Control | 1.25 µM NG52 | 2.5 µM NG52 | 5 µM NG52 | 10 µM NG52

PGK1 45kDa

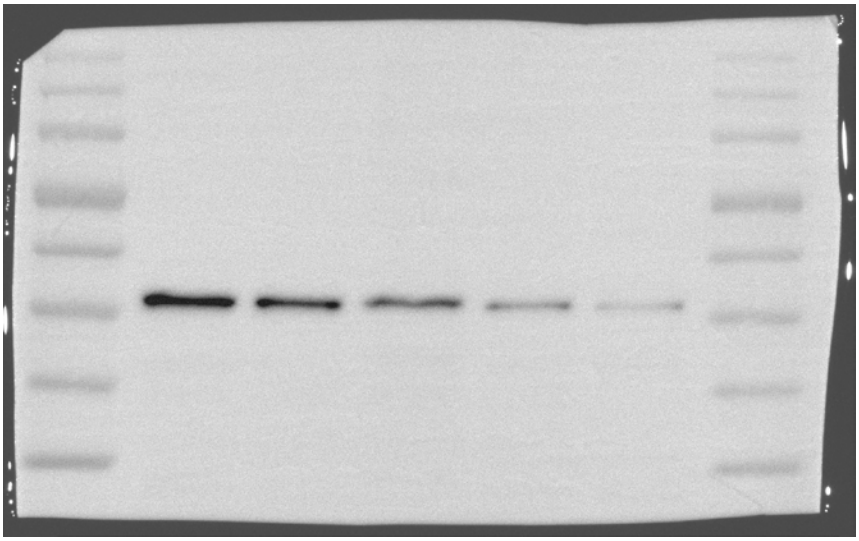

β-Tubulin 55kDa

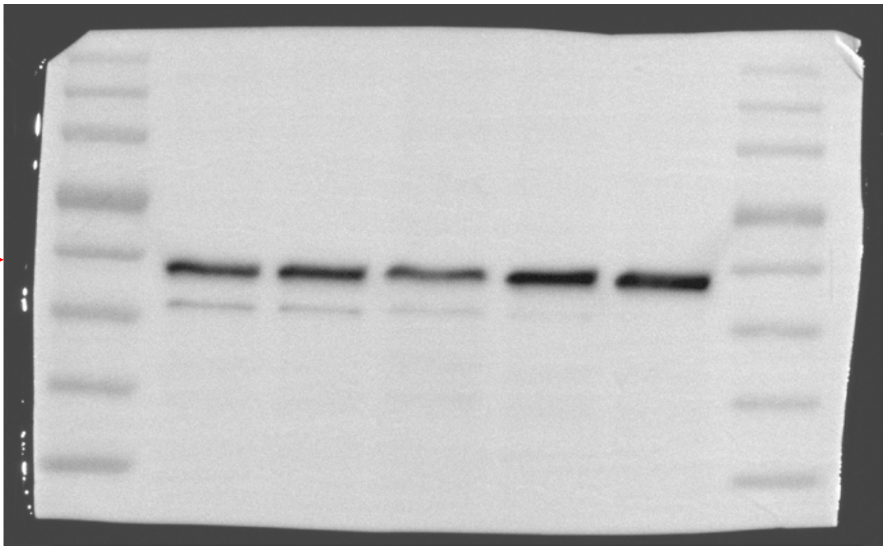

kDa

180
130
100
72
55
43
33
25

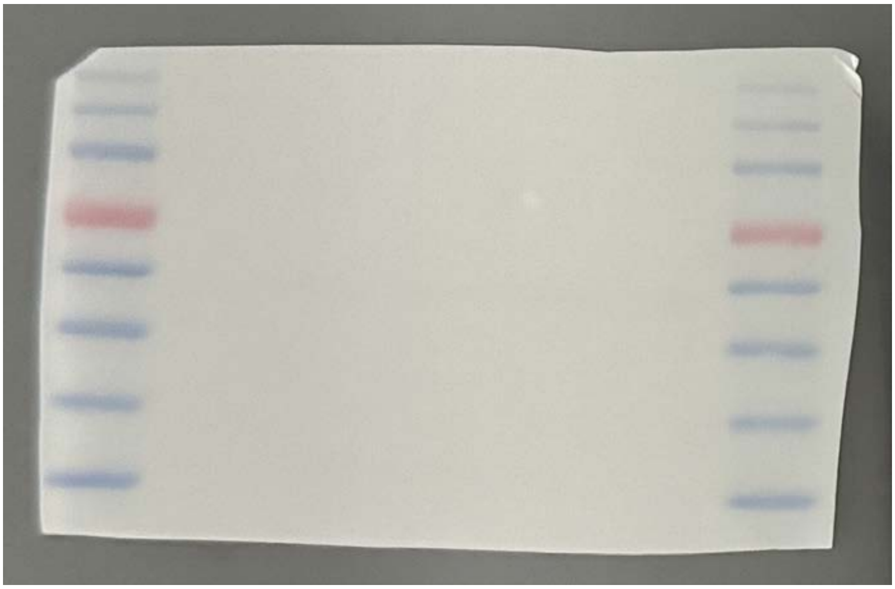

WB-48h-Test2

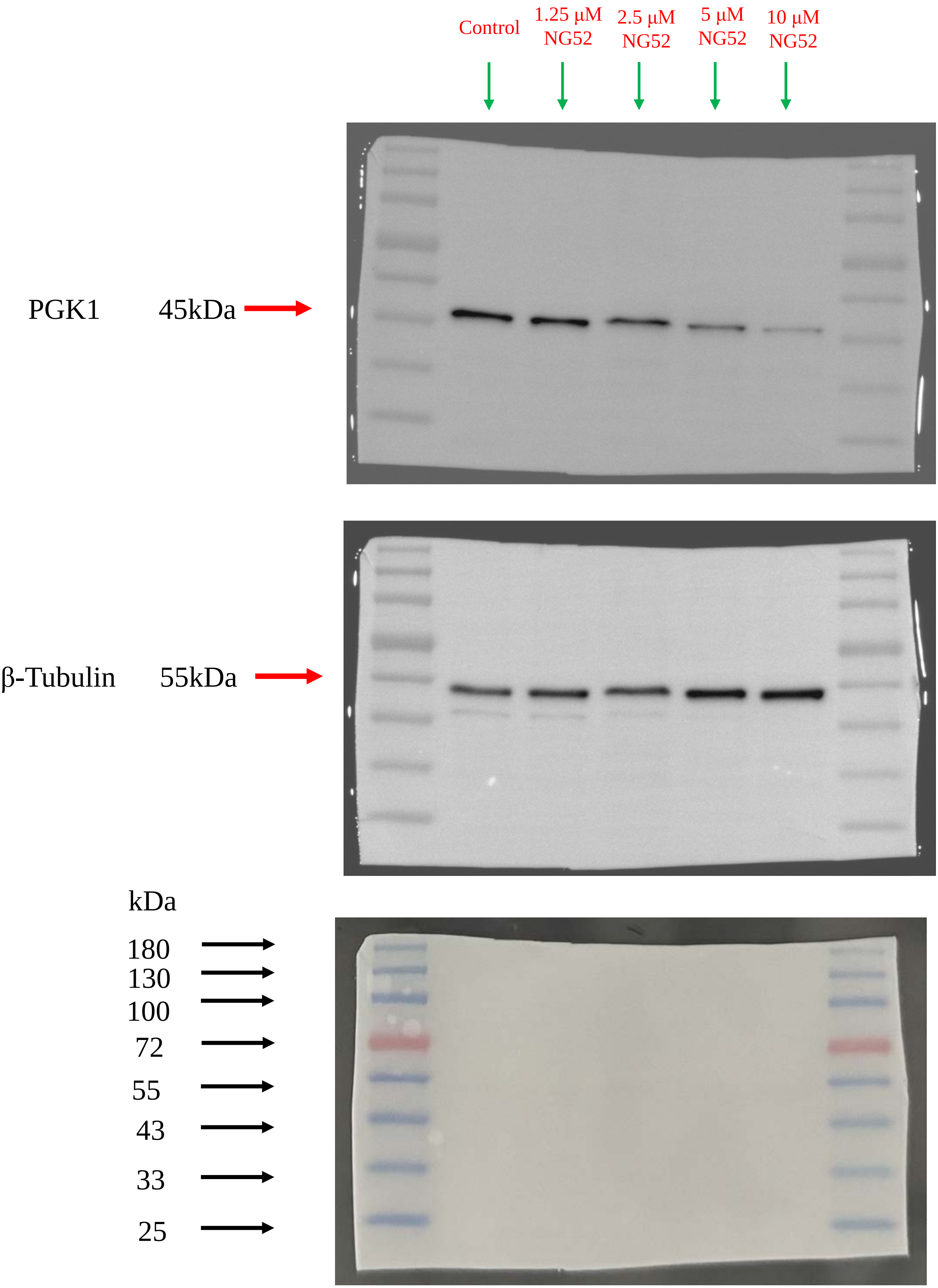
Control
1.25 μM NG52
2.5 μM NG52
5 μM NG52
10 μM NG52
PGK1
45kDa
β-Tubulin
55kDa
kDa
180
130
100
72
55
43
33
25

WB-48h-Test3

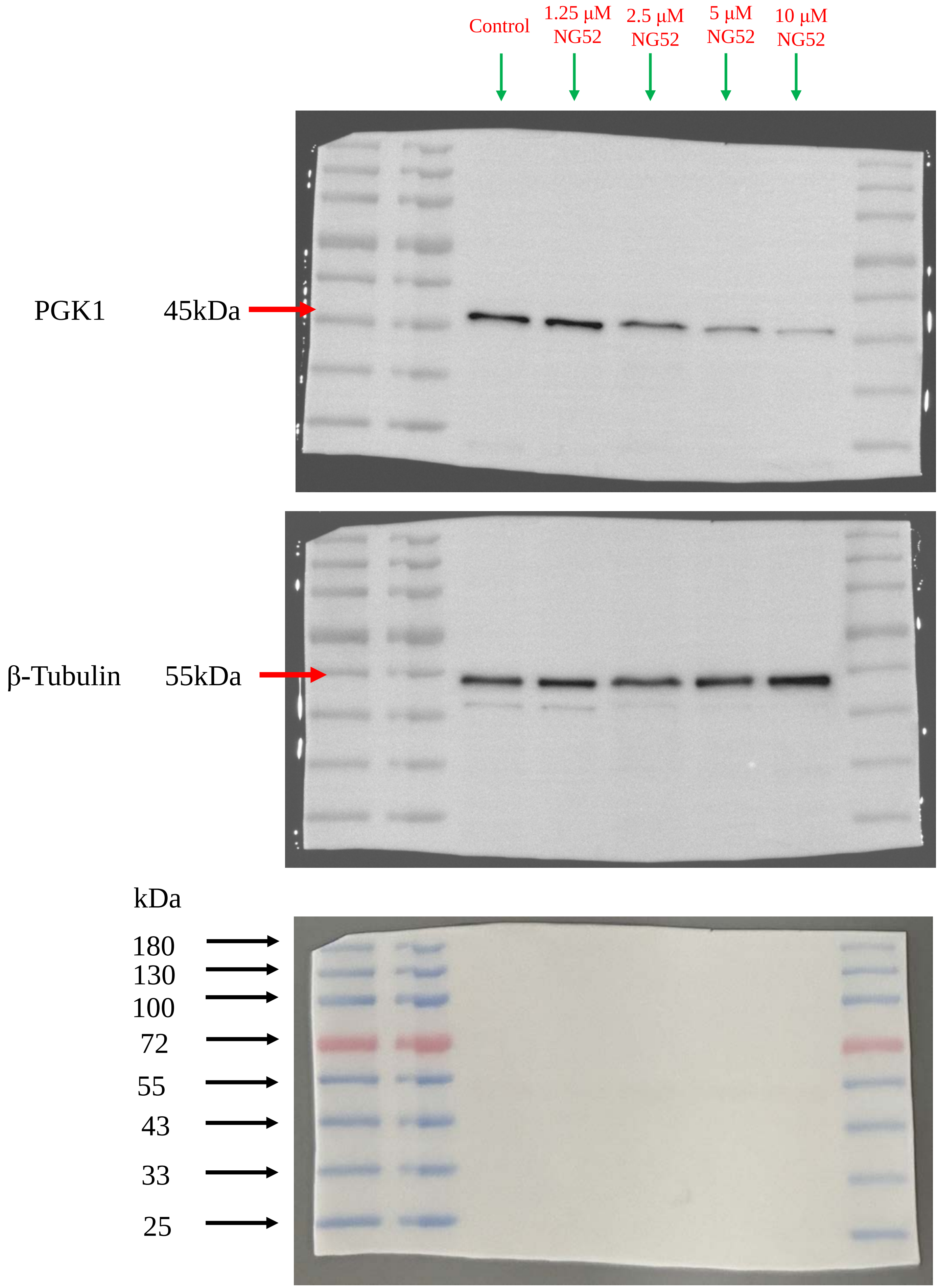
Control
1.25 µM NG52
2.5 µM NG52
5 µM NG52
10 µM NG52
PGK1
45kDa
β-Tubulin
55kDa
kDa
180
130
100
72
55
43
33
25

WB-72h-Test1

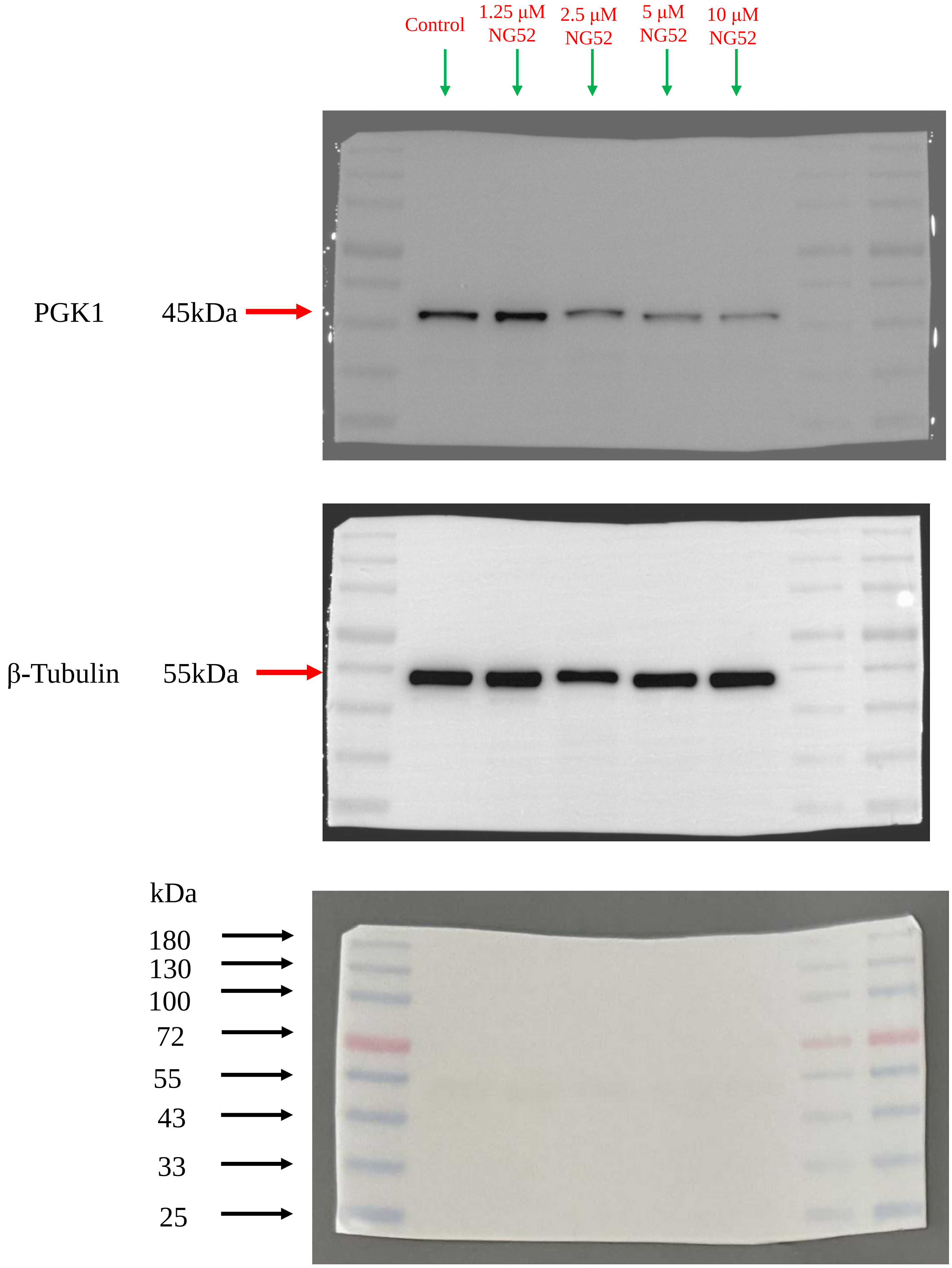
Control
1.25 μM NG52
2.5 μM NG52
5 μM NG52
10 μM NG52
PGK1
45kDa
β-Tubulin
55kDa
kDa
180
130
100
72
55
43
33
25

WB-72h-Test2

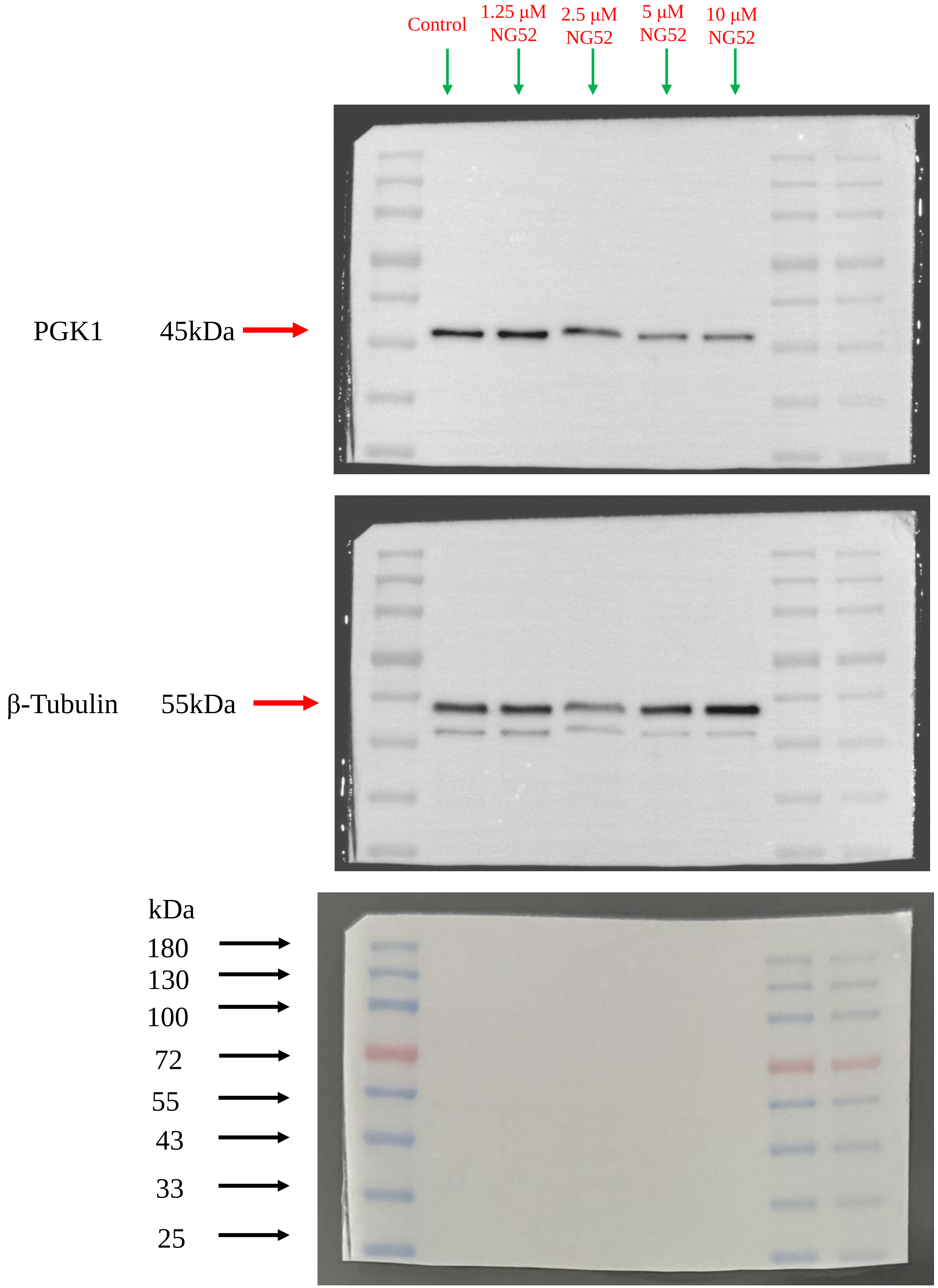

Control
1.25 µM NG52
2.5 µM NG52
5 µM NG52
10 µM NG52
PGK1
45kDa
β-Tubulin
55kDa
kDa
180
130
100
72
55
43
33
25

WB-72h-Test3

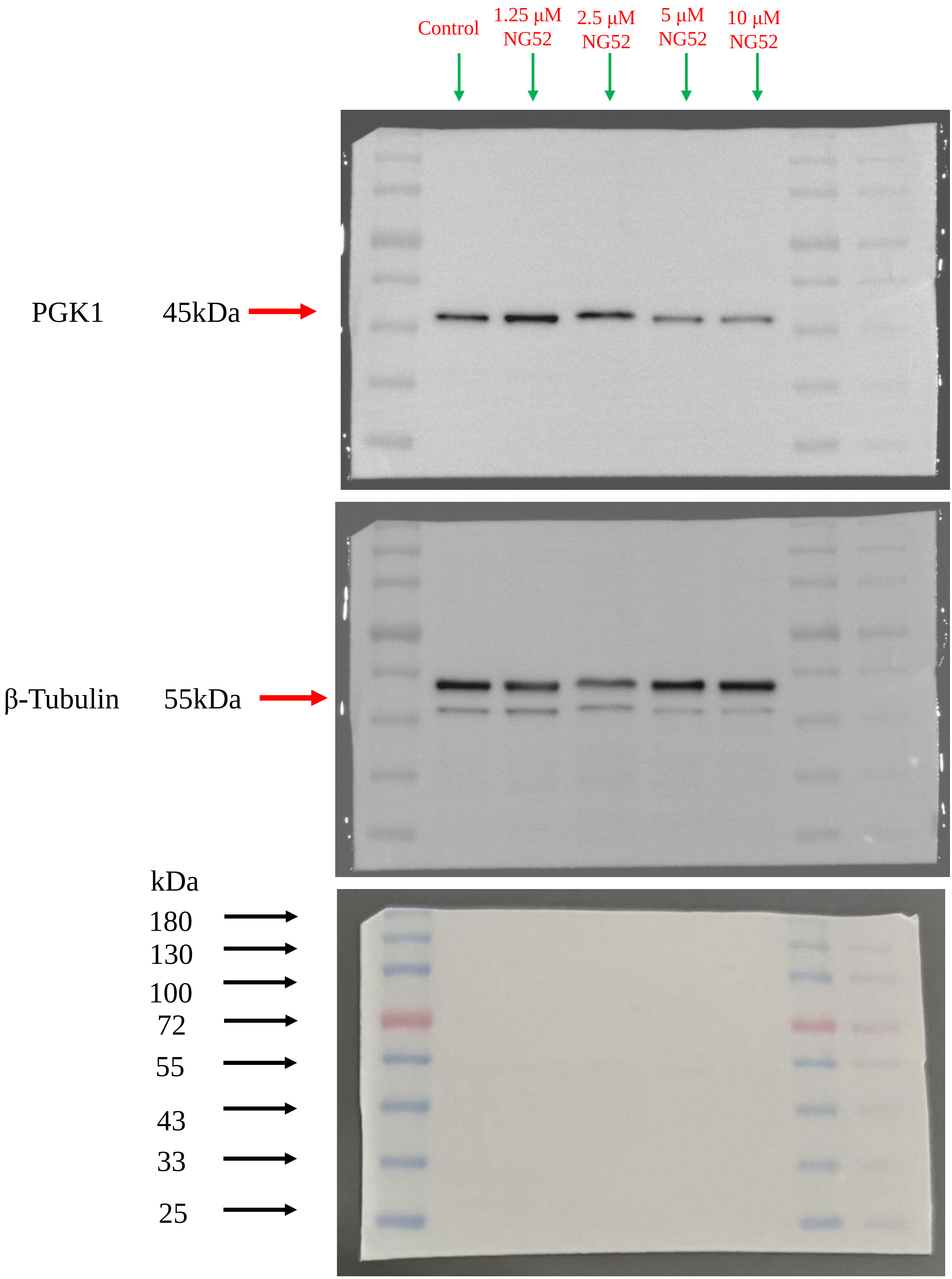

Control
1.25 μM NG52
2.5 μM NG52
5 μM NG52
10 μM NG52
PGK1
45kDa
β-Tubulin
55kDa
kDa
180
130
100
72
55
43
33
25
